# *Phrixotrix* luciferase and 6′-aminoluciferins reveal a larger luciferin phenolate binding site and provide novel far-red combinations for bioimaging purposes

**DOI:** 10.1038/s41598-019-44534-3

**Published:** 2019-06-21

**Authors:** V. R. Bevilaqua, T. Matsuhashi, G. Oliveira, P. S. L. Oliveira, T. Hirano, V. R. Viviani

**Affiliations:** 1 Graduate Program of Biotechnology and Environmental Monitoring, Federal University of São Carlos (UFSCar), Rodovia João Leme dos Santos, km 110, Itinga, Sorocaba, SP Brazil; 2 Graduate Program of Evolutive Genetics and Molecular Biology, Federal University of São Carlos (UFSCar), São Carlos, SP Brazil; 3 Brazilian Biosciences National Laboratory (LNBio), Brazilian Center for Research in Energy and Materials (CNPEM), Zip Code 13083-970, Campinas, Sao Paulo Brazil; 4 Department of Engineering Science, Graduate School of Informatics and Engineering, The University of Electro-Communications, Chofu, Tokyo 182-8585 Japan

**Keywords:** Oxidoreductases, Biological fluorescence

## Abstract

How the unique luciferase of *Phrixothrix hirtus* (PxRE) railroad worm catalyzes the emission of red bioluminescence using the same luciferin of fireflies, remains a mystery. Although PxRE luciferase is a very attractive tool for bioanalysis and bioimaging in hemoglobin rich tissues, it displays lower quantum yield (15%) when compared to green emitting luciferases (>40%). To identify which parts of PxRE luciferin binding site (LBS) determine bioluminescence color, and to develop brighter and more red-shifted emitting luciferases, we compared the effects of site-directed mutagenesis and of larger 6′-substituted aminoluciferin analogues (6′-morpholino- and 6′-pyrrolidinyl-LH) on the bioluminescence properties of PxRE and green-yellow emitting beetle luciferases. The effects of mutations in the benzothiazolyl and thiazolyl parts of PxRE LBS on the K_M_ and catalytic efficiencies, indicated their importance for luciferin binding and catalysis. However, the absence of effects on the bioluminescence spectrum indicated a less interactive LBS in PxRE during light emission. Mutations at the bottom of LBS of PxRE blue-shifted the spectra and increased catalytic efficiency, suggesting that lack of interactions of this part of LBS with excited oxyluciferin phenolate underlie red light emission. The much higher bioluminescence activity and red-shifted spectra of PxRE luciferase with 6′-morpholino- (634 nm) and 6′-pyrrolidinyl-luciferins (644 nm), when compared to other beetle luciferases, revealed a larger luciferin phenolate binding pocket. The size and orientation of the side-chains of L/I/H348 are critical for amino-analogues accommodation and modulate bioluminescence color, affecting the interactions and mobility of excited oxyluciferin phenolate. The *PxRE* luciferase and 6′-aminoluciferins provide potential far-red combinations for bioimaging applications.

## Introduction

Beetle luciferases produce bioluminescence of different colors from green to red using the same substrates, D-luciferin (LH_2_) and ATP^1,2^. Among them, railroadworms luciferases emit light with the widest range of colors, ranging from green to red. Beetle luciferases, especially firefly ones, have been extensively used in bioanalysis, and more recently in real time bioimaging of biological and pathological processes, including cancer and prospection of new drugs^3,4^. Far-red and near infra-red emitting luciferases are demanded for bioimaging such processes in bone and hemoglobin rich tissues.

Bioluminescence color is determined by the structure of the emitter and the luciferase active site microenvironment around the emitter, through non-specific polarity effects^5,6^, specific acid-base interactions^7,8^ and active site geometric and conformational effects^9^. It was unclear which part of the luciferin binding site (LBS) plays a major role in bioluminescence color determination. The tautomerization between a red-emitting keto form and yellow-green emitting enolate form of oxyluciferin on the thiazolyl part of the LBS, under assistance of a basic group, was originally proposed to play a major role^7,8,10^. Although this hypothesis was not ruled-out, more recent experimental and theoretical studies suggest that the keto form is the most likely emitter of different colors^11–15^, indicating that acid-base and electrostatic interactions of oxyluciferin phenol/phenolate groups at the bottom of LBS play a major role.

Bioluminescence color change of a luciferin-luciferase system, can also be attained by modifying the chemical structure of the luciferin affecting the spectral properties of the emitter. Several firefly luciferin analogues producing distinct bioluminescence colors were already produced^16–25^. The first active luciferin analog synthetized was 6′-aminoluciferin (NH_2_-LH), which produced red-shifted spectra with firefly luciferases, independently of pH^16^. Then, 6′-aminoluciferin (NH_2_-LH) and its 5,5-dimethyl-derivatives, 6′-amino-5,5-dimethylluciferin (NH_2_-LH-DM) and 6′-dimethylamino-5,5- dimethylluciferin (NMe_2-_LH-DM) were used as pH-insensitive probes to investigate the luciferase active site character and its interactions with the 6′-hydroxy group of oxyluciferin^22^. Other analogues having a 6′-substituted amino group with distinct electron donating character and sizes were recently synthesized, showing high bioluminescence activity with firefly luciferase and red-shifted spectra^19,21–23^. Recently, other firefly luciferin analogues producing far-red shifted and near-infrared bioluminescence were also produced^20,24,25^, including *akalumine*, which has an extended conjugation between the thiazole and benzothiazole rings in combination with a modified luciferase, and naphtyl-luciferin analogs with a mutant click beetle luciferase which produce near-infrared spectra but with low efficiency^24,25^.

Several beetle luciferases have already been cloned, sequenced and investigated^26–38^. The three-dimensional structures of firefly luciferases were solved in the absence^39,40^ and presence of luciferin-related compounds^41–43^, showing the structure of the active site in open and closed conformations^43^. Since LBS residues are invariant or conserved among beetle luciferases displaying different bioluminescence colors, no clear relationship between their identity and emission spectra was ever found^44–59^. Despite of that, several single-point mutations located either inside the LBS of firefly luciferases or outside it often resulted in red mutants^44–46,48,49,53,56^. Very recently, we showed that the electrostatic couples E311 and R337, and H310 and E354 at the bottom of the LBS constitute the pH-sensing moiety and metal binding site of firefly luciferases, and that E311 could be the critical base for green light emission^58–60^.

**Figure 1:**
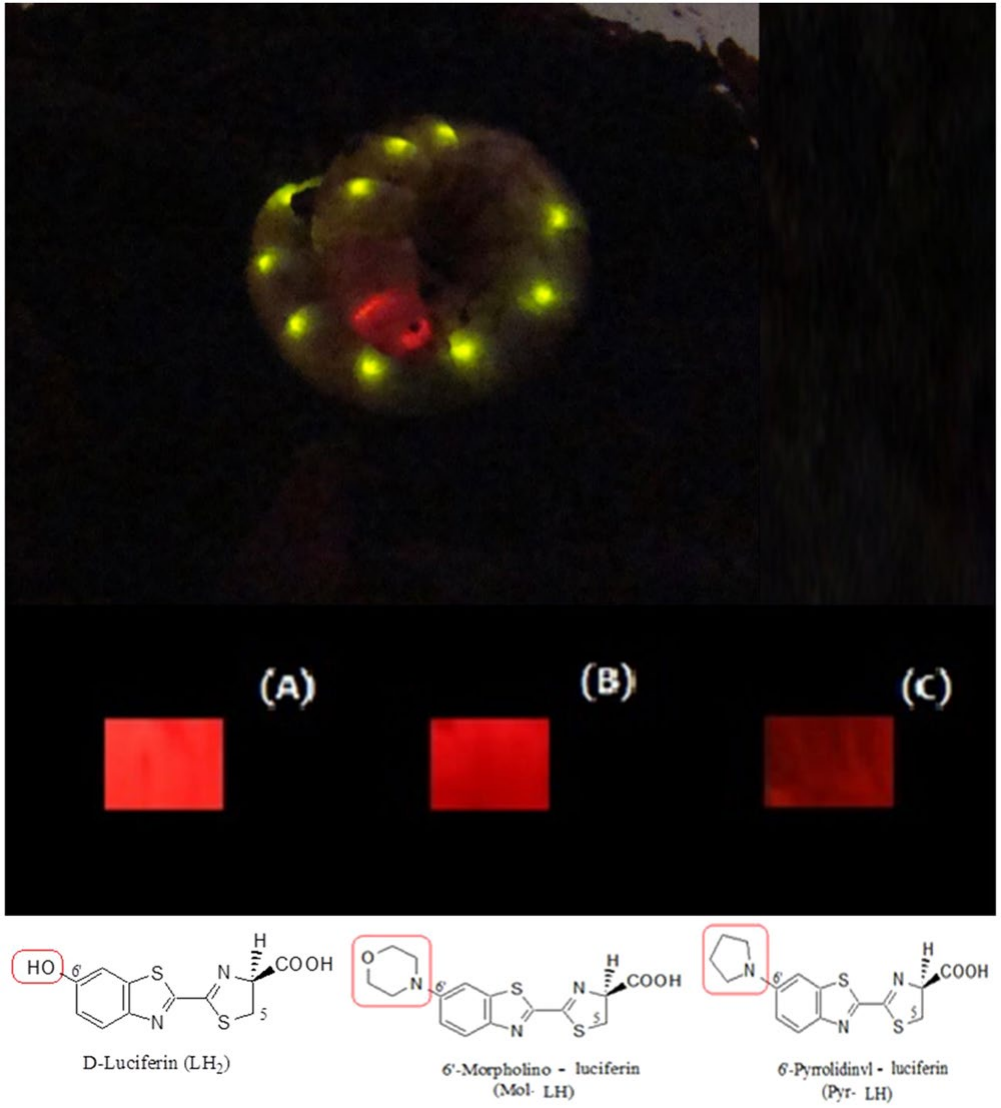
(Upper) *Phrixotrix hirtus* railroad worm and (lower) Red and far-red bioluminescence of *E. coli* expressing PxRE luciferase in presence of (**A**) D-luciferin; (**B**) 6'-morpholinoluciferin and (**C**) 6'-pyrrolidinylluciferin.

The luciferase of *Phrixothrix hirtus* (PxRE) railroad worm stands out as the only beetle luciferase that naturally emits red light (623 nm; Fig. 1), displaying one of the lowest K_M_ values for D-luciferin among beetle luciferases^34^, being potentially useful for bioanalytical assays and bioimaging in hemoglobin rich and opaque tissues such as bones. This enzyme, however, suffers from a low quantum yield^61^ and stability when compared to other green emitting beetle luciferases (40–60%), hampering its effective application in bioanalysis.

What structural features determine red light emission in PxRE luciferase remain unclear. Chimerization studies with parts of *Phrixotrix vivianii* green emitting luciferase (PxGR) and of PxRE luciferase cDNAs (RE220GR) showed that the region above residue 220 plays a major role in bioluminescence color determination. In contrast to the green emitting luciferases, however, most of the mutations, including those of the invariant/conserved active site residues R215S, H242A and A243G (R218, H245 and G246 in *P. pyralis* firefly luciferase)^47,50,52^, did not affected the bioluminescence spectrum of PxRE luciferase. The invariant active site R215 (R218 in *P. pyralis* luciferase) was found to be important for green-yellow bioluminescence in PxGR luciferase^50^, but not for red light emission in PxRE luciferase. The only mutations that affected the bioluminescence spectrum of PxRE luciferase were T226N^50^, C311T^57^ and L334R^58^ which caused modest 10–15 nm blue-shifts. The presence of the arginine at position 334 (L334 in PxRE; R337 in firefly luciferases) was found to be critical for blue-shifting the emission spectra of beetle luciferases, and its absence for red-shifting it in PxRE luciferase^58^.

With the aim to determine what structural features are responsible for red light emission in PxRE luciferase, and ultimately to develop more efficient far red-shifted emitting luciferases, here we investigated the influences of mutations in different parts of its LBS, and the effect of novel 6′-substituted amino luciferin analogues- 6'-morpholinoluciferin (Mor-LH) and 6'-pyrrolidinylluciferin (Pyr-LH)- on the bioluminescence properties of this enzyme and other green-emitting beetle luciferases.

## Results

### Background and rationale of this study

The luciferase of *P. hirtus* railroad worm is the only recombinant luciferase which naturally produces red bioluminescence among beetle luciferases, being a good starting point for developing novel far-red shifted luciferases for bioimaging purposes. However, the mechanism of red-light emission still remains to be elucidated, especially the identification of the LBS parts which are responsible for modulating bioluminescence colors in this enzyme.

Previous site-directed mutagenesis studies of PxRE luciferase showed that, in contrast to green-yellow emitting luciferases, most single-point mutation, including those in the active site (R215S, H242A, A243A), did not caused any effect on the bioluminescence spectrum^47,51,52^. The exceptions were the mutants T226N, C311T and L334R that caused 10–15 nm blue-shifts^50,57,58^. A yellow light emitting chimera (RE220GR) was produced by combining segments from residues 1–219 or PxRE luciferase and 220–545 of PxGR luciferase, indicating that the region above residue 220 plays a major role in bioluminescence color in these luciferases.

**Figure 2:**
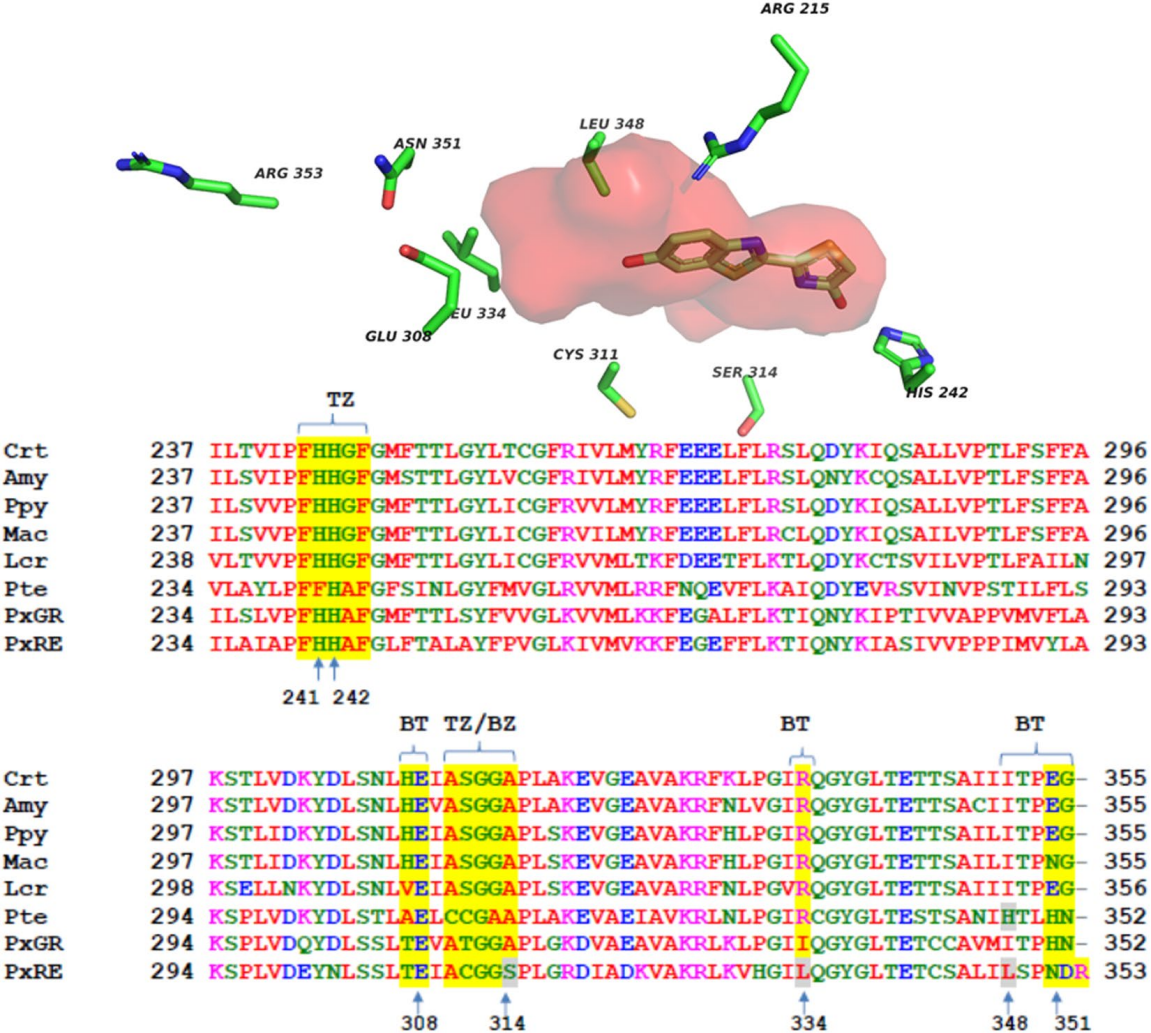
(Upper panel) threedimensional model of PxRE luciferase showing the location of the investigated residues; (Lower panel) multialignment of the luciferin binding site (LBS) segments (yellow shadow) of *Phrixotrix hirtus* red emitting luciferase with other beetle luciferases in the region between residues 238–358 (*Photinus pyralis* sequence): (BZ) Benzothiazole; (TZ) Thiazole; (BZ/TZ) Benzothiazole/Thiazole; (BT) Bottom; (red) hydrophobic residues; (green) polar residues; (blue) negatively charged residues; (pink) positively charged residue; (gray shadow) investigated residues; (Pte) *Pyrearinus termitilluminans* larval click beetle; (Mac) *Macrolampis* sp2 firefly; (Ppy) *Photinus pyralis* firefly; (Crt) *Cratomorphus distinctus* firefly; (Amy) *Amydetes vivianii* firefly; (Rol) *Ragophtalmus ohbai* starworm; (PxGR) *Phrixotrix vivianii* railroadworm green emitting and (PxRE) *Phrixotrix hirtus* railroad worm red emitting luciferase.

To investigate what part of the LBS plays a major role in red light emission in PxRE luciferase, we first investigated the effect of mutations of conserved residues in three distinct segments of the luciferin binding site (LBS) (Fig. 2) on the bioluminescence spectra and catalytic properties: (TZ: Thiazolyl side) the mutations H241F and H242K in the segment 241HHGF244. In this segment, the residue H245 in *P. pyralis* firefly luciferase (H242 in PxRE luciferase) was associated to the putative catalytic base for C4 proton abstraction, and to the stabilization of C4 carbanion and pentavalent intermediate in firefly luciferase^49,62^; (TZ/BT: between the thiazole and benzothiazole) the mutation S314T is located in the catalytic loop 311CGGS314^49^. The goal of this mutation was to analyze the effect of increased size of the side-chain on catalytic properties; (BT: Bottom) The mutation L334R was already shown to blue-shift the emission spectrum in this luciferase^58^, and the mutants N351C, N351E and R353E are located in the loop 351–360, which was already shown to influence bioluminescence colors in other beetle luciferases.

**Figure 3:**
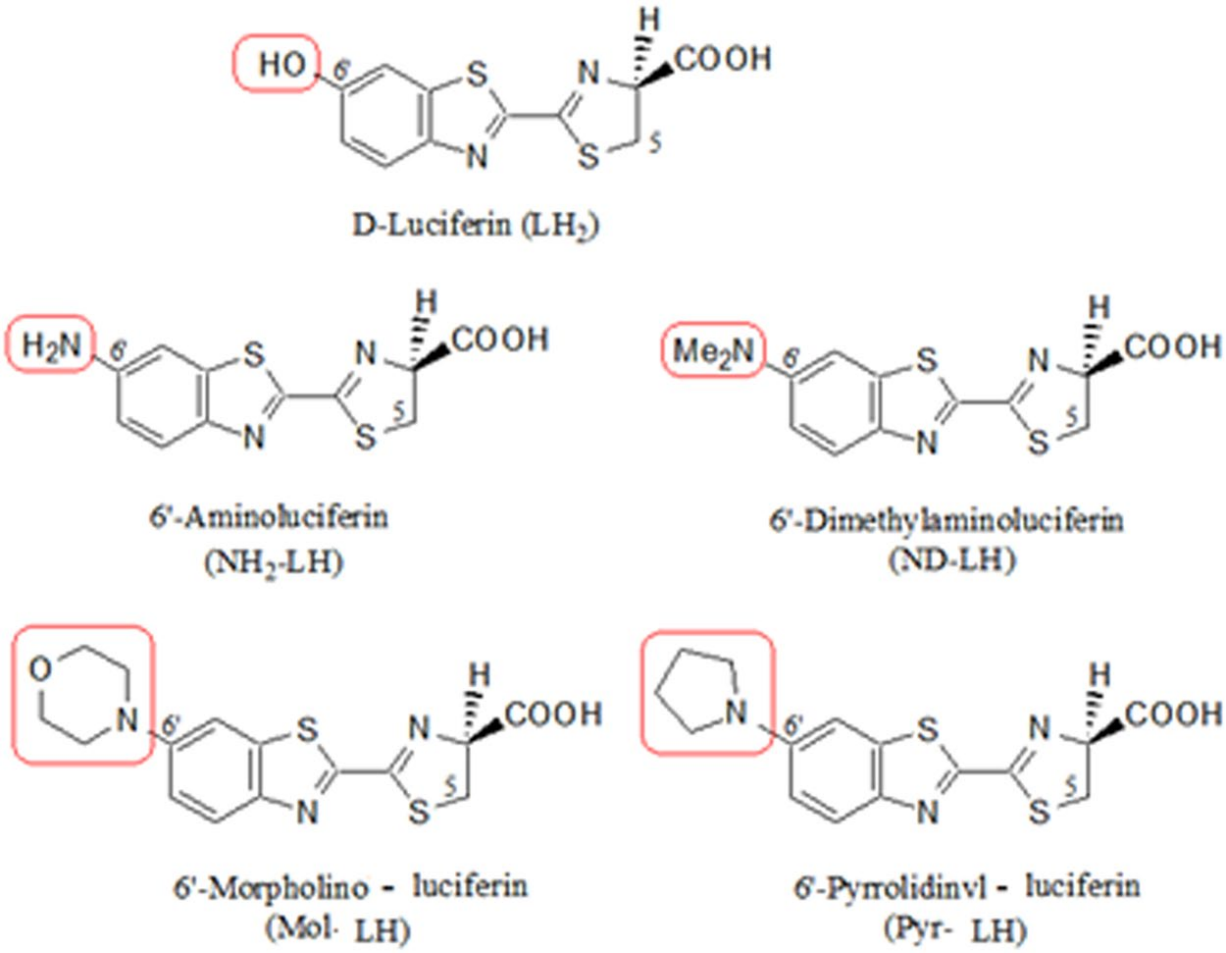
Structures of D-luciferin, 6′-aminoluciferin, and 6′-substituted amino luciferin analogues. The portions in red show the 6′-amino and substituted 6′-amino groups.

Then, to investigate the specific influence and interactions of the phenolate binding pocket at the bottom of LBS with excited oxyluciferin 6′-group, and to search novel red-shifted luciferin-luciferase combinations, we compared the effect of novel 6′-amino-analogues, in which the 6′-OH group is substituted by larger amino derivatives (Fig. 3), on the bioluminescence properties of PxRE and other green-orange emitting beetle luciferases.

### Thiazolyl and benzothiazolyl residues are important just for binding and catalysis

Most of the LBS residues, which surround the thiazolyl and benzothiazolyl moieties of luciferin were shown to be important for substrate binding and catalysis in firefly luciferase^49^. However, these residues are invariant or conserved among beetle luciferases emitting quite distinct bioluminescence colors, and therefore they are not necessarily involved in bioluminescence color determination. Despite of that, in firefly luciferases, mutations of several of these residues resulted in red-shifts or true red mutants.

As expected, the mutations in the thiazolyl and benzothiazolyl parts of PxRE luciferase LBS affected the luciferin K_M_ and catalytic efficiencies (Table 1), most of them with negative impacts, confirming their importance for luciferin binding and catalysis. Only the mutants H242K and S314T (H245 and S317 in firefly luciferase) had increased catalytic efficiencies.

**Table 1:** Summary of the effects of luciferin binding site mutations on the kinetic properties of *Phrixotrix* red-emitting luciferase and its mutants.

Luciferase	λ_max_ (nm)*	K_M_ (μM)		Specific Activity**	Oxidative Activity**	*k*_*cat*_	*k*_ox_	k_*cat*_/K_M_		k_*ox*_/K_M_	
	[Half-Bandwidth]	ATP	LH_2_	(10^9^c*ps*/mg) [Relative Activity]		(10^−6^*cps*)	(10^−6^*cps*)	ATP	(LH_2_)	ATP	(LH_2_)
WT	626 [82]	230	7	70[100]	65[93]	8.3	7.8	0.04	1.2	0.034	1.11
H241F	622 [82]	43	56	43[61]	45[64]	4	4.2	0.1	0.073	0.098	0.075
**H242K**	**629 [79]**	**23**	**5**	**65[93]**	**98[140]**	**6.22**	**9.32**	**0.28**	**1.3**	**0.414**	**1.86**
**S314T**	**626 [75]**	**424**	**3**	**80[114]**	**100[142]**	**7.8**	**9.7**	**0.02**	**2.61**	**0.023**	**3.2**
**L334R**	**612 [80]**	**22**	**6**	**160[229]**	**217[310]**	**14**	**20**	**0.65**	**2.42**	**0.96**	**3.56**
**L348H**	**608[89]**	**37**	**10**	**134[191]**	**96[137]**	**12.7**	**9.2**	**0.34**	**1.2**	**0.25**	**0.87**
N351C	622 [79]	3.7	0.7	8.1[11]	15[21]	0.769	1.42	0.2	1.09	0.38	2
N351E	621 [79]	136	82	57[81]	20[29]	5.5	2	0.04	0.066	0.014	0.024
R353E	626 [74]	16	11	2[3]	1.5[2]	0.85	0.34	0.053	0.077	0.021	0.03

*The estimated peak error is ±2.5 nm.

**The standard deviations of activity averaged 25% and ranged from 0.4 to 45%.

### Influence of mutations on the overall bioluminescence and oxidative activities

**Figure 4:**
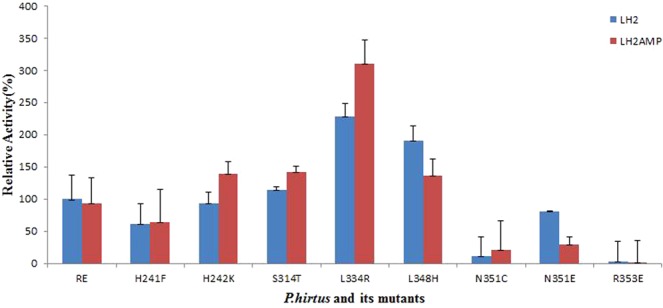
Relative overall and oxidative activities of *P. hirtus* red-emitting luciferase and its mutants: (red) overall bioluminescent activity starting with ATP and luciferin; (Blue) oxidative bioluminescent activity starting with luciferyl-adenylate.

We first compared the overall bioluminescence activity starting with LH_2_ and ATP, and the oxidative activity starting the reaction with luciferyl-adenylate (LH_2_AMP), the product of the first-half reaction which is oxidized producing light (Fig. 4). Previously, we compared the oxidative activity of beetle luciferases and *Zophobas* luciferase-like enzyme, and found that most luciferases, with the exception of *Pyrearinus termitilluminans* (Pte) click beetle, displayed similar oxidative activity to the overall bioluminescence activity^63^.

As expected, most of the PxRE mutants had decreased specific activities in relation to the wild-type (WT) enzyme (Table 1). For the mutants N351C and R353E, both the overall and oxidative activities were severely impacted. On the other hand, the mutants S314T, L334R and L348H (which will be discussed later) had higher specific activity. For the mutants H241F, S314T e R353E, the ratios of overall bioluminescence activity/oxidative activity were close to 1.0, indicating that the mutations did not impacted specifically the oxidative activity. For the mutants H242K e L334R the ratio was <1 indicating that the oxidative activity was increased, whereas for the mutants L348H and N351E the ratio was >1, indicating that they impacted the oxidative activity.

### Effect on the K_M_ values for ATP and LH_2_

The K_M_ value of wild type enzyme for ATP was estimated as 230 µM^34^. All the mutants, with exception of S314T, showed decreased K_M_ values for ATP (Table 1). Regarding the K_M_ values for luciferin, the mutants H242K and S314T, which are located on the thiazolyl side (TZ), and between the thiazolyl and benzothiazolyl sides (TZ/BT) of the LBS, respectively, and the mutants L334R and N351C which are located at the bottom (BT) of the luciferin binding site, had slightly decreased K_M_ values for luciferin. Among them, the mutants S314T and N351C displayed the lowest K_M_ values for luciferin. Because N351E and R353E decreased the affinity for luciferin, and N351C had opposite effect, it is likely that insertion of negative charges in this critical loop impacts the luciferin binding.

### Effect on the catalytic constants and efficiencies

Because the values of Vmax for luciferase luminescent reaction are given in *cps* (counts for second), and not in photon/s, the absolute values of ***k***_**cat**_ and *k*_ox_ in s^−1^ could not be determined, and therefore the values were reported in cps (counts for second). Although these values are not absolute, they can be safely used as relative values of catalytic constants for the luciferases compared in this study.

Most of the mutants had considerably changed values of catalytic constants and efficiencies (Table 1). The mutants N351E, N351C and R353E were the less efficient among all these mutants. However, the mutants H242K, S314T and L334R had increased catalytic efficiencies for luciferin: S314T and the blue-shifted L334R mutants doubled the value of the catalytic efficiencies for luciferin in relation to the wild-type. In L334R, the increase of catalytic constant accounted for most of the increase in catalytic efficiency, whereas in S314T the increase of affinity for luciferin accounted for the higher value of catalytic efficiency.

The invariant residue H245 in *P. pyralis* firefly luciferase, corresponding to H242 of PxRE luciferase, was already shown to have catalytic role in the stabilization of the pentavalent intermediate in the thiazolyl part of the luciferin binding site, and possibly as a putative base abstracting C4 proton and stabilization of carbanion during the oxidative step^49,64^. It was also shown to display important function in stereoselection of the bioluminescent substrate, D-luciferin, against L-luciferin^65^. Noteworthy, the substitution H242K in PxRE luciferase increased the catalytic efficiency mainly by increasing the oxidative catalytic constant indicating that lysine at this position influences the oxidation reaction. The insertion of a permanent positive charge of lysine may help to stabilize the C4 carbanion during the oxygenation step increasing the oxidative step.

The mutation S314T, despite being conservative, substituted a smaller residue by larger one, slightly decreasing the cavity size near the luciferin benzothiazole and thiazoline rings, contributing to increase the affinity for luciferin and probably to stabilize of luciferin near other catalytic groups responsible for oxidative reaction, increasing the catalytic efficiency.

### PxRE displays a less interactive LBS during light emission

Despite the kinetic effects, none of the above mutations (H241F, H24K, S341T) affected bioluminescence spectrum of PxRE luciferase (Table 1), similarly to the previously published LBS mutations (R215S, H242A, A243G)^47,50,51^. The absence of effect of most mutations on the bioluminescence spectra of PxRE luciferase, in sharp contrast to other green-yellow emitting luciferases in which the corresponding mutations result in red-shifts or even red mutants^47–50,52^, indicated that the interactivity of these residues is important for green light emission, but not for red light emission.

Because the emissive step occurs just after the oxidative step, the lack of effect of most LBS mutations on the bioluminescence spectrum of PxRE luciferase indicate a lack of interactions of LBS with excited oxyluciferin during light emission. This is in agreement with non-enzymatic studies with luciferyl-adenylate chemiluminescence which showed that red chemiluminescence requires a less structured and relaxed microenvironment than green chemiluminescence^63^. A less interactive LBS is also supported by studies with the fluorescent probes TNS, ANS and amino-analogs, which showed a considerably more polar LBS for PxRE luciferase in relation to other yellow-green emitting luciferases^22,52^.

### The bottom of LBS is critical for red light emission

Noteworthy, the only mutations that affected bioluminescence color in PxRE luciferase (T226N, C311 and L334)^50,58,60^, are located at or near the bottom of the LBS, indicating that this part of LBS is indeed critical for modulating bioluminescence colors. Interestingly, the blue-shift effects that only these mutations caused, indicate that they somehow may help to establish novel interactions between the bottom of LBS and excited oxyluciferin phenolate, increasing the energy of its excited state. This agrees with our recent results with firefly luciferases showing that pH and heavy metals also bind to this side of the LBS, modulating their bioluminescence colors^58,60^.

The mutation L334R, for example, was previously shown to blue-shift the spectrum of PxRE luciferase by establishing a salt bridge with E308 (E311 in *P. pyralis* firefly luciferase) and likely an electrostatic interaction with excited oxyluciferin phenolate in green-yellow emitting luciferases^58,60^. The new formed interaction between E308 and R334 in PxRE luciferase, may close and squeeze the bottom of the LBS pushing the oxyluciferin toward other catalytic groups, contributing for the higher catalytic efficiencies, mainly the oxidative one. Very recently, Hall *et al*.^25^ also obtained a red emitting luciferase (617 nm) upon the mutation R334S in a click beetle luciferase, reinforcing our previously proposed hypothesis about the importance of R334 for green light emission in beetle luciferases^58–60^.

### Bioluminescence of 6′-substituted amino analogues with beetle luciferases

Considering the importance of the bottom of LBS in bioluminescence color determination, we decided to further investigate the specific influence of the oxyluciferin phenol/phenolate binding part of the LBS, and its interactions with the 6′ group of oxyluciferin in bioluminescence color modulation of PxRE and beetle luciferases, ‘using 6′-substituted amino-analogues. We compared the bioluminescence properties of PxRE luciferase, its mutants including the yellow-emitting chimera RE220GR, and other recombinant green-orange emitting beetle luciferases available in our laboratory with 6′-amino- (NH_2_-LH), 6′-morpholino- (Mor-LH) and 6′-pyrrolidinyl-luciferin (Pyr-LH) analogues.

### Bioluminescence activity of beetle luciferases with 6′-amino-analogs

**Figure 5:**
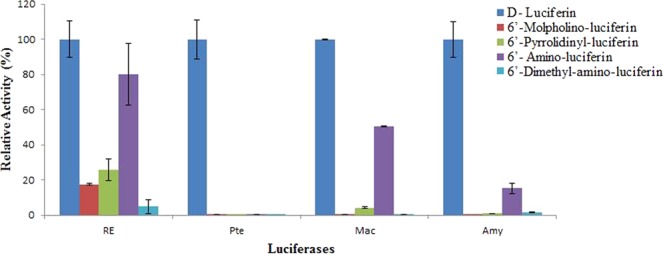
Relative activity of beetle luciferases with firefly D-luciferin and 6′-amino-analogues: *Macrolampis* sp2 and *Amydetes vivianii* firefly, *P. termitilluminans* click beetle and *P. hirtus* railroadworm. The activities of each luciferases were normalized for D-luciferin.

When comparing the activity of different amino analogues in relation to LH_2_ (Table 2; Fig. 5), NH_2_-LH was the substrate which usually gave the highest relative activity with different luciferases.

**Table 2:** Bioluminescent activities of beetle luciferases and *Phrixotrix hirtus* red-emitting luciferase mutants with firefly D-luciferin and 6′-amino-analogues.

Luciferase	Activity (10^9^cps/mg)* [Relative Activity]**				
	LH_2_	NH_2_-LH_2_	NMe_2_-LH	Mor-LH	Pyr-LH
**Firefly**					
*Amydetes viviani*	130[100]	45[35.2]	1.2[1]	0.83[0.6]	6.6[5.2]
*Cratomorphus*	37[100]	1.7[4.6]	0.02[0.05]	2.9[0.008]	3.4[0.9]
*Macrolampis*	200[100]	100[50.6]	0.4[0.18]	0.6[0.3]	8[4.3]
**Click beetle**					
*Pyrearinus termitilluminans*	200[100]	0.6[0.4]	0.03[0.02]	0.2[0.09]	0.05[0.03]
H348L	78.5[100]	0.07[0.09]	0.03[0.04]	0.014[0.017]	0.005[0.0061]
*Pyrophorus angustus*	51[100]	0.45[0.09]	0.015[0.03]	0.4[0.007]	0.06[0.1]
**Railroadworm**					
*Phrixothrix vivianii*	230[100]	3.5[15.3]	0.33[1.43]	0.02[0.082]	0.21[0.9]
RE220GR	12[100]	1[8]	0.057[0.45]	0.08[0.64]	0.34[2.7]
*Phrixothrix hirtus* (WT)	70[100]	56[80]	3.4[4.84]	12[17.6]	18[25.7]
R215S	2.2[100]	0.54[24]	0.016[0.7]	0.026[1.2]	0.3[12.8]
H241F	43[100]	25[54]	1.7[3.6]	6.6[14.1]	14[30]
H242K	52[100]	84[162]	0.004 [0.007]	0.07[0.1]	0.09[0.2]
S314T	21.5[100]	69[32]	0.009[0.004]	3.2[1.5]	20[9.2]
L334R	160[100]	82[60.8]	3[2.2]	11[8.3]	55[41]
L348H	134[100]	18[10.8]	0.8[0.5]	6.4[4]	4.4[2.7]
N351C	8.1[100]	54[64.5]	0.61[0.7]	4.1[4.5]	16[19]
N351E	57[100]	52[9]	0.49[0.8]	1.6[2.9]	2.80[5]
R353E	2[100]	90[45]	0.08[0.4]	0.073[0.4]	1.5[7.4]

*The standard deviations of specific activities averaged 6.5% and ranged from 1 to 62%.

**Relative activities in relation to firefly luciferin (LH_2_).

Noteworthy, among all beetle luciferases, PxRE luciferase was the one which comparatively gave the highest luminescence activity with most 6′-substituted amino analogues in relation to D-luciferin, especially larger 6′-substituted amino ones (Mor-LH and Pyr-LH), showing 18–26% of the activity observed with wild-type luciferin (Fig. 5, Table 2). The closer PxGR gave much lower activity (0.082–0.9%) with such analogues in relation to luciferin. Firefly luciferases, which emit green-yellow light and are pH-sensitive, gave intermediate values (0.008–5.2%). Finally, the *Pyrearinus termitilluminans* green emitting (Pte) and *Pyrophorus angustus* orange emitting click beetle luciferases were those which gave the lowest activities (0.007–0.1%).

### Bioluminescence activity of *PxRE* luciferase mutants with 6′-amino-analogues

**Figure 6:**
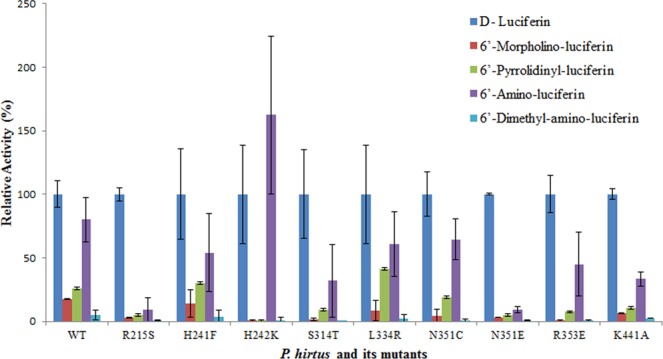
Relative activity of *P. hirtus* red-emitting luciferase and mutants with 6′-amino-analogues and luciferin. The activities of each mutant luciferase were normalized for D-luciferin.

The wild-type PxRE luciferase was in general more active than its mutants with most amino analogues (Fig. 6; Table 2). Only the mutant H242K showed 62% higher activity with NH_2_-LH in relation to luciferin. The mutant L334R was the most active with Pyr-LH (41%), followed by the mutants H241F (30%), wild-type luciferase (26%), N351C (19%), S314T (9%). The mutants H241F and L334R gave comparable results to the wild-type luciferase with regard to Mor-LH (Fig. 6; Table 2). However, these differences must be seen with some caution, since the standard error was relatively high, which was likely caused by the relative instability of these amino-analogs.

### Bioluminescence spectra of 6′-amino-analogues with beetle luciferases

Previously we showed that NH_2_-LH produced red-shifted spectra with most green-yellow emitting beetle luciferases in relation to LH_2_, and blue-shifted emission with PxRE luciferase, being a reasonable probe to investigate the active site microenvironment polarity^22^. Other 6′-substituted amino analogues also resulted in red-shifted spectra with green-yellow emitting beetle luciferases.

**Figure 7:**
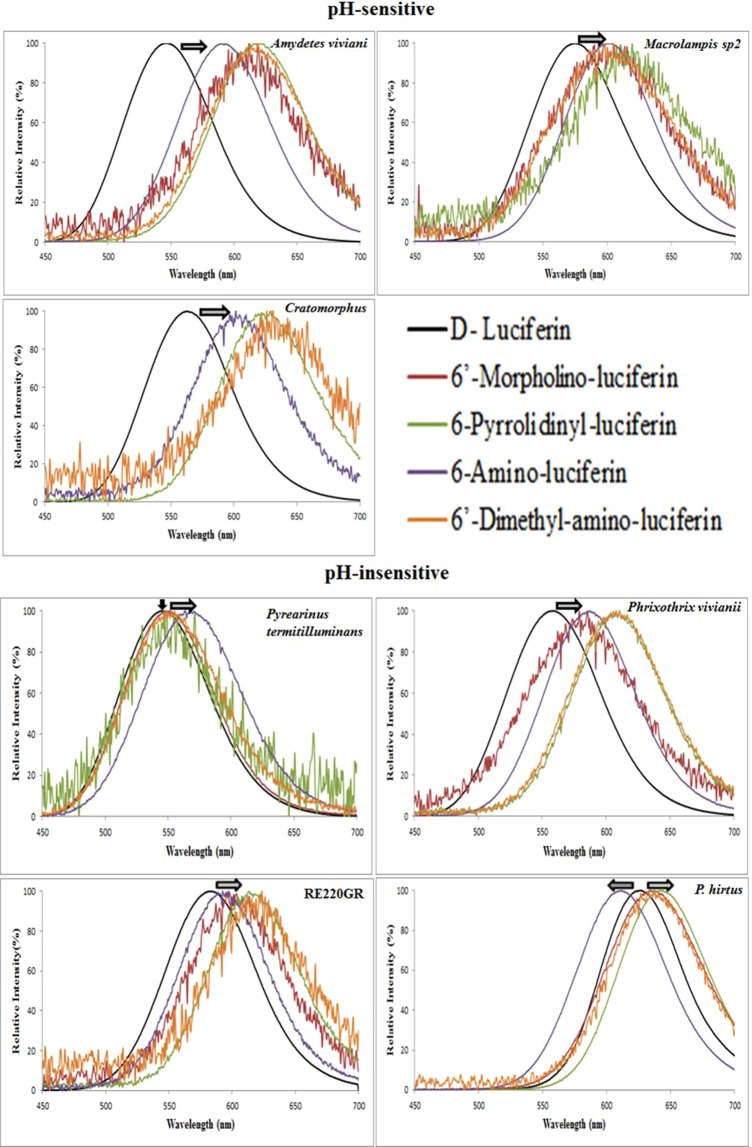
Bioluminescence spectra of beetle luciferases with 6′-amino-analogues.

Similarly, here we showed that the novel 6′-substituted amino analogues, Pyr-LH and Mor-LH, also produced red-shifted spectra with most green-yellow emitting luciferases in relation to LH_2_ (Fig. 7; Table 3), independently of pH-sensitivity, which is expected by the electron donating effect of the substituents in more polar environments. The green-emitting luciferase of *Pyrearinus termitilluminans*, displayed only small red shift with such analogs indicating a lees hydrophobic environment. In contrast, PxRE luciferase and most of its mutants, displayed blue-shifted spectra with NH_2_-LH, indicating a more hydrophobic environment for this analog, and red-shifted spectra with all larger amino analogues indicating more polar environments for the latter analogues. In the case of Pyr-LH and Mor-LH analogues, these batchromic shifts were larger than 20 nm in relation to the wild-type luciferase with firefly luciferin.

**Table 3:** Bioluminescence spectra of beetle luciferases and *Phrixotrix hirtus* red emitting luciferase mutants with firefly luciferin and 6′-amino analogues.

Luciferase	λ_max_ [Half-Band] (nm)*				
	LH_2_	NH_2_-LH_2_	NMe_2_-LH	Mor-LH	Pyr-LH
**Fireflies**					
*Amydetes viviani*	547[85]	591[90]	618[95]	612[104]	615[95]
*Cratomorphus*	563[85]	601[79]	626[107]	^#^	629[97]
*Macrolampis*	575[86]	602[83]	605[110]	597[114]	619[107]
**Click beetle**					
*Pyrearinus termitilluminans*	546[87]	563[92]	549[93]	551[84]	548 [89]
H348L	559	^#^	^#^	^#^	^#^
*Pyrophorus angustus*	591[78]	^#^	^#^	^#^	^#^
**Railroadworms**					
*Phrixothrix vivianii*	558[89]	587[86]	608[91]	579[109]	611[87]
RE220GR	584[86]	593 [86]	624[90]	597 [92]	614 [89]
*Phrixothrix hirtus* (WT)	626[82]	612 [87]	638[86]	634[92]	644[84]
R215S	630 [80]	620 [86]	651 [80]	647 [94]	654[87]
H241F	622[82]	608[89]	634[87]	631[99]	638[88]
H242K	629 [79]	614[86]	640[86]	634 [86]	622 [91]
S314T	626[75]	612[85]	^#^	626[92]	639[87]
L334R	613 [80]	608[85]	631[89]	621[94]	628[85]
L348H	608[89]	613[89]	640[85]	635[91]	646[74]
N351C	622[79]	611 [83]	640[99]	628[92]	642 [85]
N351E	621 [79]	614 [84]	636[89]	635[90]	638 [90]
R353E	626[74]	608 [82]	640[84]	635[94]	639[83]

*The estimated peak error is ±2.5 nm. Above 620 nm the estimated error was ±3 nm.

^#^The much lower activities did not allow to measure the bioluminescence spectra.

### Bioluminescence spectra of *Phrixotrix* luciferase mutants with 6′-amino analogues

**Figure 8:**
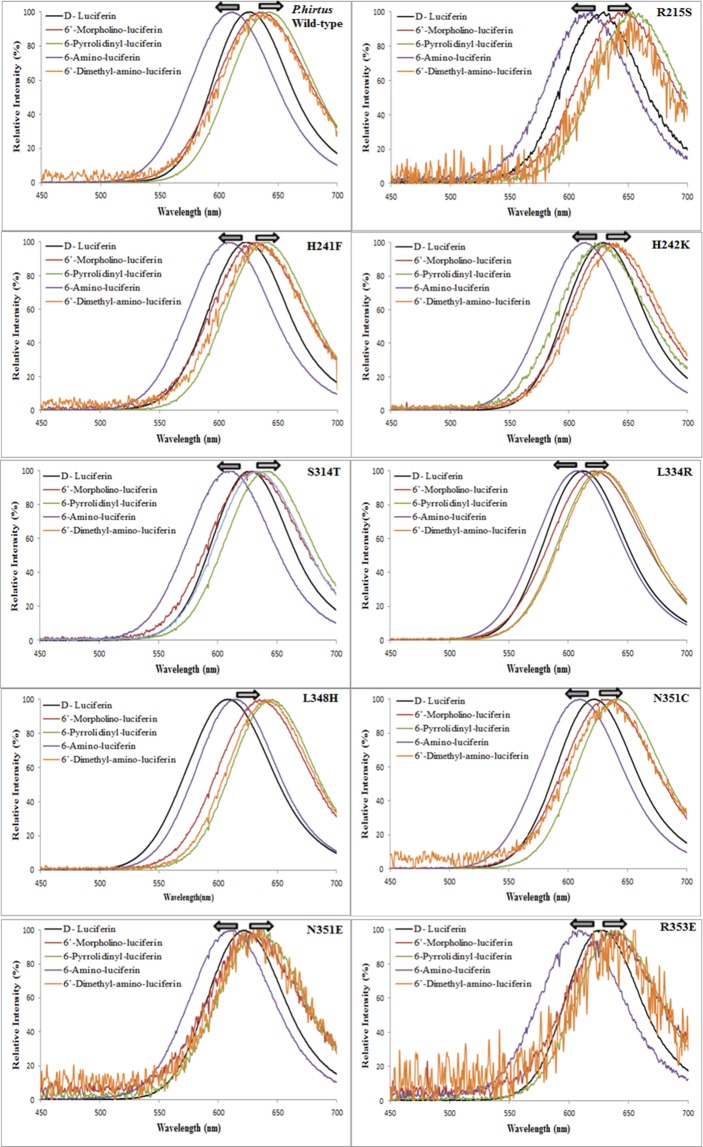
Bioluminescence spectra of *P. hirtus* red emitting luciferase and its mutants with LH_2_, NH_2_-LH, and 6′-substituted amino analogues.

Similarly to the wild-type enzyme, most of the PxRE mutants displayed blue-shifts with NH_2_-LH in relation to LH_2_, whereas most of the mutants displayed similar red-shifts with all larger amino analogues in relation to LH_2_ (Fig. 8; Table 3). The exception was H242K with Pyr-LH, which displayed a modest 7 nm blue-shift in relation to LH_2_. Despite small, this shift represents a significant inversion of the emission spectral behavior in relation to the wild-type and other mutants, indicating a different interaction. Finally, the mutant R215S in PxRE luciferase, despite being much less active and displaying a similar bioluminescence spectrum to the wild-type luciferase with LH_2_^47^, displayed the largest red-shifts (~30 nm) with such analogues.

### Amino-analogues and modelling reveal a larger luciferin phenolate cavity in PxRE luciferase

**Figure 9:**
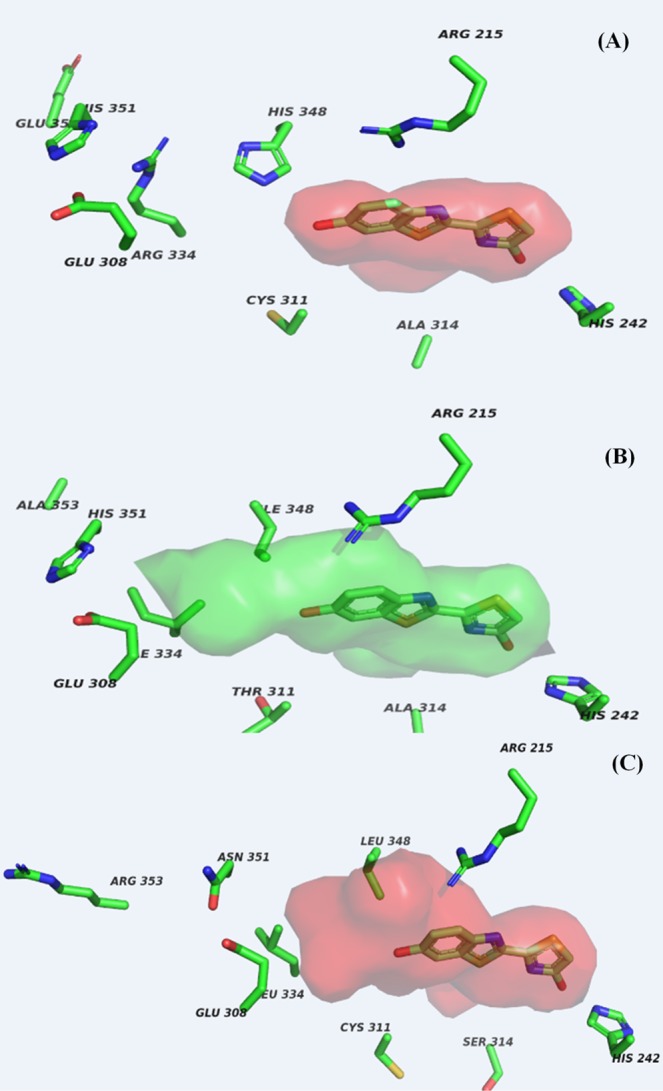
Active site modelling of beetle luciferases showing a larger phenol binding cavity in *Phrixotrix hirtus* red emitting luciferase: (**A**) *Pyrearinus termitilluminans* luciferase; (**B**) *Phrixotrix vivianii* luciferase; (**C**) *P. hirtus* red-emitting luciferase.

Considering the higher activity (Fig. 5) and red-shifted spectra of PxRE luciferase and mutants with substituted amino analogues at the 6′ position in relation to other beetle luciferases, and that the mutations affecting bioluminescence spectra were located at the bottom of the LBS, we compared the structures of the corresponding part of the LBS pockets of beetle luciferase (Fig. 9) to find out structural characteristics that may explain such differential properties of PxRE luciferase with amino analogues in relation to other beetle luciferases.

Modelling the three-dimensional structures of three pH-insensitive luciferases (*Pyrearinus termitilluminans*; *Phrixotrix vivianii* and *P. hirtus*) emitting distinct colors based on the threedimensional structure of *Luciola cruciata* firefly luciferase complexed with the luciferyl-adenylate analogue 5′-*O*-[(*N*-dehydroluciferyl)-sulphamoyl]-adenosine (DLSA) as the template^43^, showed the PxRE luciferase displays a larger cavity around the phenol group in the LBS (Fig. 9). In this region, multialignment and modelling studies showed that PxRE luciferase has L348 (Fig. 2), whereas in PxGR, which has much lower activity with various amino analogues, the corresponding position is substituted by I348 whose side-chain is sterically bent closer to the phenolate group. In contrast, in the click beetle luciferases, independently of the bioluminescence color, the position 348 is always substituted by the His (H348) whose large imidazole side-chain obstructs this cavity. Furthermore, in PxRE luciferase the close invariant guanidinium side-chain of R215 (R218 in *P. pyralis* firefly luciferase) is rotated away from oxyluciferin phenolate contributing to open the cavity, whereas in PxGR the side-chain of R215 is bent closer to the oxyluciferin phenolate. Therefore, PxRE displays a larger 6′-phenolate binding pocket which may better accommodate large 6′-substituted amino luciferin analogues, resulting in higher bioluminescence activity when compared to other beetle luciferases (Fig. 5). Besides L348, the size and orientation of the side-chains of residues R215 and L334 also contribute for a larger cavity.

### Position 348 is critical for amino-analogues accommodation and bioluminescence colors

**Figure 10:**
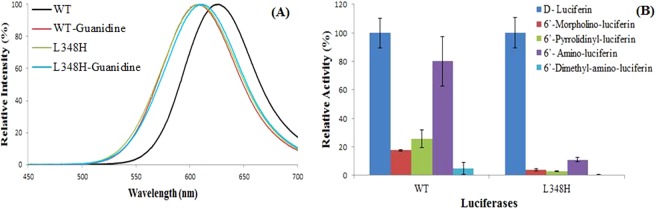
(Left panel) Bioluminescence spectra of *Phrixotrix* red-emitting luciferase mutant L348H in the presence and absence of guanidine; (Right panel) Bioluminescence activity with LH_2_, NH_2_-LH, and 6′-substituted amino analogues. The activities of each mutant luciferase were normalized for D-luciferin.

Considering that natural substitutions at position 348 were the main ones affecting the size of the oxyluciferin phenolate binding cavity, we investigated whether mutation at this position affect bioluminescence colors and activity for amino-analogues. In PxRE luciferase, we replaced L348 by histidine, which is found in click beetle luciferases which display the lowest activities among beetle luciferases with amino-analogues. The activity of L348H mutant with these large amino analogs in relation to luciferin was indeed considerably lower (~ 4%) than the wild-type luciferase (Fig. 10). Finally, the mutation L348H resulted in a large 20 nm blue-shift, leading to an orange emitting mutant (Fig. 10). On the other hand, the reverse mutation H348L in *P. termitilluminans* click beetle luciferase considerably decreased both the activity with either D-luciferin and 6′-aminoluciferin analogues, and caused a ~15 nm red-shift with luciferin. These results indicate that the size and orientation of the side-chain at position 348 is critical for amino-analog accommodation. The smaller L348 side-chain in *P. hirtus* red emitting luciferase is critical for the accommodation large 6′-substituted analogues, explaining the higher bioluminescence activity of *P. hirtus* luciferase in relation to other beetle luciferases (Fig. 10). On the other hand, the large imidazole side-chain of H348 in click beetle luciferases hampers the accommodation of the large amino substituents of the analogues.

The large blue-shift of the bioluminescence spectrum upon the mutation L348H, in PxRE, and the 15 nm red-shift upon the reverse mutation H348L in *P. termitilluminans* green emitting luciferase, also indicates that the position 348 is critical for bioluminescence color determination in pH-insensitive beetle luciferases. The frequency variations (cm^−1^), which reflect the energy changes associated with the spectral shifts caused by both mutations, had similar values but in opposite directions (PxRE Δ*F*_L348H_ = 47 cm^−1^; and *P. termitilluminans* luciferase Δ*F*_H348L_ = −47 cm^−1^), giving an indication that these mutations indeed display antagonistic effects, as it would be expected.

### Effect of guanidine on the bioluminescence spectra

We also tested the effect of guanidine, which is long known to blue-shift the spectrum of the wild-type PxRE luciferase and most of its mutants^47,50^, by simulating the effect of the lack of a critical arginine at position 334 (L334 in PxRE)^58^. Most beetle luciferases display arginine at the corresponding position (R337), which is apparently important for establishing a salt bridge with E311 closing the active site, and for establishing an electrostatic interaction with excited oxyluciferin^58^, favoring green light emission. However, guanidine did not cause any effect on the bioluminescence spectrum of the mutant L348H (Fig. 10), indicating that this mutation affects the binding of guanidine.

### Influence of cavity size on bioluminescence color determination

The results shown here indicate that the cavity size could be a main factor influencing bioluminescence color determination. The more red-shifted spectra of such analogs is consistent with the higher electrodonating properties of the 6′-substituents and with a larger cavity which allows higher mobility with loss of vibrational energy. In the larger cavity observed in PxRE luciferase, there is more space for water molecules and for excited oxyluciferin mobility, polarizing the environment and explaining the lack of interactions of LBS residues with excited oxyluciferin phenolate during light emission. On the other hand, in a smaller cavity, which is found in green-yellow emitting beetle luciferases, tighter interactions are expected between excited oxyluciferin phenolate and the active site groups.

The differential effects of amino-analogues and of the guanidine on the bioluminescence spectra of PxRE mutants in relation to the wild-type luciferase provide important clues about the LBS interactions with excited oxyluciferin and the underlying mechanism of bioluminescence color determination in beetle luciferases.

Both the lack of guanidine blue-shifting effect and the anomalous red-shift with amino-luciferin in the mutant L348H, also support for the existence of a larger cavity in the phenolate binding pocket of PxRE luciferase. The anomalous small red-shift (~5 nm) with NH_2_-LH in the mutant L348H when compared to other mutants and wild-type luciferase which usually display >10 nm blue-shifts with these two analogs, may indicate the establishment of a novel interaction between the imidazole side-chain of H348 with NH_2_-LH, which is otherwise absent in the larger cavity of the wild-type enzyme. In the case of guanidine, which is known to blue-shift the emission spectra in PxRE luciferase and most of its mutants by simulating a lost arginine (L334 in PxRE and the respective R337 in firefly luciferase), the lack of blue-shifting effect in the mutant L348H indicates that the larger side chain of histidine may exclude guanidine from this cavity, replacing its interaction with oxyluciferin phenolate.

The side-chains of R215 and V284, and the peptide bond of C311, whose mutations were previously shown to be important for bioluminescence colors in different beetle luciferases^43,47,57^ also contribute for making a larger cavity in PxRE luciferase and the differential effect of specific interactions of LBS with 6′-substituted amino groups.

The invariant R215 was previously shown to be important for green bioluminescence in PxGR and *Photinus pyralis* (R218) firefly luciferase, but not for red emission in PxRE luciferase^47,50^. We suggested that, somehow, the invariant R215 side-chain could be displaced from oxyluciferin phenolate in PxRE luciferase. Modelling studies indeed showed that the R215 side chain is rotated away from oxyluciferin phenolate in PxRE luciferase in relation to the closer PxGR, contributing for a larger cavity in the former luciferase. On the other hand, in the closer relative PxGR, the rotation of R215 side chain and the substitution of L348 by isoleucine contribute to reduce the size of this cavity. The considerably red-shifted spectra of the mutant R215S with larger 6′-substituted amino analogues, suggest that the side-chain of the residue at position 215 may establish an interaction with the larger 6′-substituted amino groups of the analogues, which is otherwise absent with smaller analogues or with luciferin.

**Figure 11:**
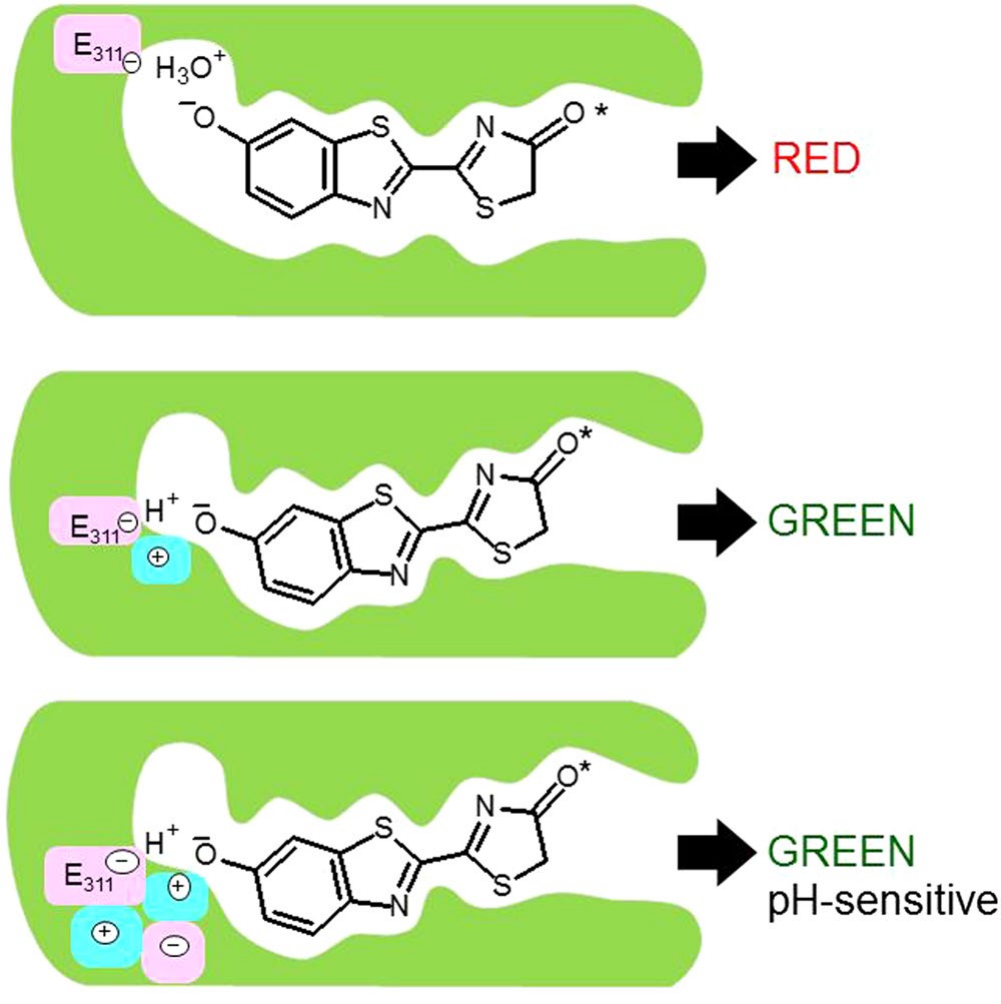
Proposed mechanism of bioluminescence color modulation by the active site of PxRE and green-emitting luciferases.

Taken together, our results confirm the importance of the bottom of the LBS in bioluminescence color determination in beetle luciferases, and indicate that the size of the oxyluciferin phenolate binding pocket plays a critical role, especially for red-light emission. In the case of pH-sensitive firefly luciferases, the pH and heavy metals were shown to affect the salt bridges E311/R337 and H310/E354 which electrostatically close the bottom of the LBS^58–60^, modulating the rigidity and size of this cavity during light emission, and the ability to retain the excited oxyluciferin released proton near its phenolate group into a high energy state (Fig. 11). In the case of the unique PxRE luciferase, there is a naturally larger and polar phenol binding cavity in this portion of the LBS, reducing the specific interactions between excited oxyluciferin phenolate with the LBS, and the ability to retain the excited oxyluciferin released proton near its phenolate, promoting red light emission (Fig. 11). On the other hand, in pH-insensitive green-yellow-emitting luciferases, this cavity is filled up by larger side-chains excluding water, increasing the specific acid-base and electrostatic interactions with excited oxyluciferin, retaining excited oxyluciferin released proton near its phenolate, blue-shifting the emission spectra.

Therefore, the unique structure of PxRE luciferase active site has evolved toward a compromise between two factors: (I) the tight accommodation of both the thiazole and benzothiazole parts of the luciferin inside the LBS to efficiently promote the adenylation and oxidative reactions, increasing the light emission, and (II) an increase of the cavity size of the phenol binding pocket, relaxing and polarizing the environment around the phenolate group of excited oxyluciferin producing red light.

Whereas this manuscript was under preparation, the first crystallographic threedimensional structure of PxRE luciferase^66^ was solved, revealing that this enzyme has a unique octameric quaternary structure. However, no relationship between quaternary structure and bioluminescence color was found, since the monomeric form also produced red light. Although the manuscript also reported additional site-directed mutagenesis of residues which were already investigated by our research group, no additional insight could be obtained about bioluminescence color mechanism. Therefore, we believe that this manuscript brings a new insight about mechanism of red light emission in the enigmatic PxRE luciferase.

### Potential Far-red shifted combination for bioimaging purposes

We showed that PxRE luciferase, its mutants, and 6′-substituted amino analogues produce far-red shifted bioluminescence (>640 nm) with relatively high activity when compared to other beetle luciferases. This can also be demonstrated *in vivo* with *E. coli* colonies expressing the PxRE luciferase (Fig. 1). Whereas combinations emitting in the near-infrared (664 to 758 nm) were already obtained using naphtyl-luciferin analogs and modified click-beetle luciferase^20,24,25^, the light output signal of such combinations is still 5000 to 10,000 times lower when compared with luciferin^25^. Although PxRE luciferase and modified 6′-aminoluciferin analogs still emit in a shorter wavelength region than the system using naphtyl-luciferin or akalumine, it provides a more efficient far-red shifted combination. Furthermore, this system has the potential for further development of more active and more far-red shifted (>640 nm) combinations suitable for real-time bioimaging in hemoglobin and melanin rich tissues.

## Material and Methods

### Plasmids and beetle luciferases cDNAs

The cDNAs for *Phrixotrix hirtus* red-emitting (PxRE)*, Pyrearinus termitilluminans* green emitting (Pte) and *Macrolampis* firefly (Mac) luciferases were previously subcloned into pCold-vector (Takara). The cDNA for *Phrixotrix vivianii* green emitting luciferase (PxGR) was subcloned in pCAN vector. The cDNA of *Amydetes vivianii* (Amy) and *Pyrophorus angustus* (Pang) luciferases were cloned in pSport vector (Invitrogen) and the cDNA of *Cratomorphus distincus* luciferase (Crt) in pBluescript vector (Agilent)^35,38,57,58,61^.

### Site-directed mutagenesis

The mutants H241F, H242K, S314T, L348H, N351C, N351E, R353E and K441A were obtained by site-directed mutagenesis using a Agilent site-mutagenesis kit (Catalog 200518). The plasmids containing the luciferase cDNAs were amplified using *Pfu* turbo polymerase and 2 complementary primers containing the desired mutation, using a thermal cycler (1 cycle 95 °C; 25 cycles 95 °C, 30 s; 55 °C, 1 min and 68 °C 7 min). After amplification, mutated plasmids containing staggered nicks were generated. The products were treated with *Dpn* I in order to digest non-mutated parental plasmids, and used directly to transform *E. coli* XL1-Blue cells. The following forward and respective reverse primers were used (the mutation codon is highlighted in bold): (RE-H241F) GCC CCT **TTC** TTC CAC GCC TTC, (RE-H242K) GCC CCT TTC CAC **AAA** GCC TTC, (RE-S314T) C TGT GGC GGC **ACC** CCT CTG GG, (RE-L348H) GCC CTG ATC **CAC** AGC CCC AAC G, (RE-N351C) CCTG AGC CCC **TGC** GAT AG, (RE-N351E) CTG AGC CCC **GAG** GAT AGA GAG C, (RE-R353E) CCC AAC GAT **GAA** GAG CTG AAG A, (RE-K441A) GAG CTC ATC **GCG** TAC AAG GGC.

### Luciferase expression and purification

For luciferase expression, transformed *E. coli* BL21-DE3 cells were grown in 100–1000 mL of LB medium at 37 °C up to OD_600_ = 0.4, and then induced at 18 °C with 0.4 mM IPTG during 18 h. Cells were harvested by centrifugation at 2,500 g for 15 min and resuspended in extraction buffer consisting of 50 mM sodium phosphate buffer, 300mM NaCl, 10mM imidazol and protease inhibitor cocktail (Roche), pH 7.0, lysed by ultrasonication and centrifuged at 15,000 g for 15 min at 4 °C. The N-terminal histidine-tagged PxRE, PxGR, Pte and Mac luciferases were further purified by agarose-Nickel affinity chromatography followed by dialysis and anion-exchange chromatography, according to established procedures^53,54^. The concentrations of purified luciferases were between 0.5 and 1 mg/mL, and the estimated purity, according to SDS-PAGE gels were about 90%.

### Measurement of luciferase activity

Luciferase bioluminescence intensities were measured using an AB2200 (ATTO; Tokyo, Japan) luminometer. The assays were performed by mixing 5 μL of 40 mM ATP/80 mM MgSO_4_ with a solution consisting of 5 μL of luciferase and 85 μl of 0.5 mM luciferin in 0.10 M Tris-HCl pH 8.0 in a luminometer tube. All measurements were done in triplicate for at least three independent luciferase preparations, and averages and the standard deviations were reported in the figures.

### Kinetics measurements and K_M_ determination

The K_M_ assays for luciferin were performed by mixing 5 μL of 40 mM ATP, 80 mM MgSO_4_ in a solution containing 10 μL of luciferase, 75 μL of 0.10 M Tris-HCl (pH 8.0) and luciferin at final concentrations between 0.01 and 1 mM. The K_M_ assays for ATP were performed by mixing 5 μL of 80 mM MgSO_4_ in a solution containing 10 μL of luciferase, 75 μL of 0.10 M Tris-HCl (pH 8.0) and ATP at final concentrations in the range of 0.02 to 2 mM. Both assays were performed in triplicate. The K_M_ values were calculated using Lineweaver-Burk plots taking the peak of intensity (I_0_) as a measure of V_0_. All measurements were done in triplicate and averages were reported.

### Luciferyl-adenylate synthesis

The luciferyl-adenylate was prepared using acidic D- luciferin and AMP by following a previously described preparation procedure for D-luciferyl-adenylate^63^. Luciferyl-adenylate was analyzed with silica gel TLC (moving phase: ethyl acetate/ethanol/water (5:3:2), followed by revelation by fluorescence with a UV lamp. Luciferyl-adenylate displayed yellowish fluorescence with an R_f_ = 0.68 (R_f(luciferin)_ = 0.87 with greenish fluorescence). A luciferyl-adenylate concentration was estimated from stoichiometric amounts of luciferin and ATP used for its synthesis. According to such estimations, luciferyl-adenylate concentration in stock solution are in the range between 5 to 10 mM.

### Determination of *k*_cat_ and *k*_ox_

The overall ***k***_**cat**_ and *k*_ox_ were determined by calculating the ratio of luminescence activities in *cps* by the number of luciferase molecules based on the specific bioluminescence activities measured with luciferin and ATP (overall *k*_cat_), and with luciferyl-adenylate (*k*_ox_), respectively^64,65^. Because the absolute value of *cps* in photon/s could not be determined, the absolute values of ***k***_**cat**_ and *k*_ox_ in s^−1^ could not be determined, and therefore the values were reported in cps (counts for second). Although these values are not absolute, they can be safely used as relative values of catalytic constants.

### 6′-Substituted amino analogues

All the 6′-substituted amino luciferin analogues (Fig. 3) were synthetized as reported previously^19,21,23^. Stock solutions of 10 mM were prepared in DMSO and kept at −20 °C in the dark.

### Bioluminescence spectra

Bioluminescence spectra reported here were recorded in ATTO Lumispectra spectroluminometer (Tokyo, Japan) with cooled CCD camera For the *in vitro* bioluminescence recorded using the spectroluminometer, 5.0 μL of luciferases were mixed with 90 μL of 0.10 M Tris-HCl pH 8.0, 5 μL of specific substrate (10 mM D-luciferin; luciferyl-adenylate or 6′-aminoluciferin analogues), and 5 μL of 40 mM ATP/80 mM MgSO_4_. The bioluminescence spectra were measured in triplicate for at least three independent luciferase preparations. The estimated peak error was ± 2.5 nm. Above 620 nm we assumed peak errors of ±3 nm.

### Homology modelling

Homology-based models of *Phrixotrix hirtus*, *Pyrearinus termitilluminans, Amydetes vivianii* and *Macrolampis sp2*. luciferases were constructed using as template the three-dimensional structure of *Luciola cruciata* luciferase in the presence of 5′-O-[(N-dehydroluciferyl)-sulphamoyl]-adenosine (DLSA) (PDB code 2D1S) and of oxyluciferin and AMP (PDB code – 2D1R) as previously described^67–69^ using an automated homology modelling protocol YASARA software^67^. Visualization and analyses of the best model of each luciferase were performed using PyMol version 1.4.1^67^. Volume and rendering of luciferin pocket was carried out using KVFinder^67^.

### Concluding remarks

Site-directed mutagenesis effects on kinetic constants confirm that the thiazole and benzothiazole parts of the luciferin binding site (LBS) are important for luciferin binding and catalysis of PxRE luciferase. However, the lack of effect of such mutations on the bioluminescence spectrum of PxRE luciferase, when compared to other green-yellow emitting beetle luciferases, indicate that the LBS becomes less interactive during the light emitting step. The only mutations affecting the bioluminescence spectrum of this luciferase were located at the bottom of the LBS and caused blue-shifts, indicating that the lack of interactions of this part of LBS with excited oxyluciferin phenolate underlie red light emission. The much higher bioluminescence activity and the more red-shifted spectra of PxRE luciferase with amino analogues with large 6′-substituents in relation to other green-light emitting beetle luciferases, indeed reveal a larger luciferin phenol binding cavity, where the side-chain size and orientation of residue 348 plays a major role for 6′-substituted amino-analogues accommodation and for bioluminescence color determination. In PxRE larger cavity, excited oxyluciferin phenol group has higher mobility releasing its acidic proton with lower energy to the surrounding water molecules, resulting in red light emission. In green-yellow light emitting luciferases, smaller cavities promote better and more rigid interactions with excited oxyluciferin phenolate, retaining excited oxyluciferin released proton near its phenolate into a high energy state, blue-shifting the emission spectra. The higher bioluminescence activity and more red-shifted spectra of PxRE luciferase with 6′-substituted amino-analogues provide new far-red shifted combinations potentially useful for bioimaging applications.
